# Role of TIM-1 in the development and treatment of tumours

**DOI:** 10.3389/fcell.2024.1307806

**Published:** 2024-05-20

**Authors:** Jinmeng Cao, Jilin Qing, Liya Zhu, Zhizhong Chen

**Affiliations:** ^1^ Joint Inspection Center of Precision Medicine, The People’s Hospital of Guangxi Zhuang Autonomous Region and Guangxi Academy of Medical Sciences, Nanning, Guangxi, China; ^2^ School of Clinical Medicine, Guilin Medical University, Guilin, Guangxi, China; ^3^ Center for Reproductive Medicine and Genetics, The People’s Hospital of Guangxi Zhuang Autonomous Region and Guangxi Academy of Medical Sciences, Nanning, Guangxi, China; ^4^ Graduate school, Guangxi University of Chinese Medicine, Nanning, Guangxi, China

**Keywords:** TIM-1, tumour, target, the tumour microenvironment, therapy

## Abstract

T-cell immunoglobulin and mucin structural domain 1 (TIM-1, also known as hepatitis A virus cell receptor 1) is a co-stimulatory molecule that is expressed predominantly on the surface of T cells. TIM-1 promotes the activation and proliferation of T cells, cytokine secretion, and can also be overexpressed in various types of cancer. Upregulation of TIM-1 expression may be associated with the development and progression of cancer. After reviewing the literature, we propose that TIM-1 affects tumour development mainly through two pathways. In the Direct pathway: overexpression in tumours activates tumour-related signaling pathways, mediates the proliferation, apoptosis, invasion and metastasis, and directly affects tumour development directly. In the indirect pathway: In addition to changing the tumour microenvironment and influencing the growth of tumours, TIM-1 binds to ligands to encourage the activation, proliferation, and generation of cytokines by immune cells. This review examines how TIM-1 stimulates the development of tumours in direct and indirect ways, and how TIM-1 is exploited as a target for cancer therapy.

## 1 Introduction

The human T-cell immunoglobulin and mucin structural domain (TIM) gene family, which consists of TIM-1, TIM-3, and TIM-4, is located on chromatin 5q33.2. Protein expression of the TIM-1 (also known as kidney injury molecule-1and hepatitis A virus cell receptor), is increased on the surface of injured renal epithelial cells. This protein, which is expressed primarily on T helper type 2 (Th2) cells, is a crucial component in the development of allergies and asthma. The tumour microenvironment has been demonstrated to be improved by TIM-1, which is also expressed on cluster of differentiation (CD)8^+^ T cells, natural killer (NK) cells, macrophages, dendritic cells (DCs), B cells, and mast cells. TIM-1 has been shown to increase the immune response indirectly by encouraging cytokine production and enhancing the activity of T cells (Du et al., 2016; Kong et al., 2020). Liu et al. (2018) revealed that high methylation of cg07320595 was strongly related with longer overall survival (OS) and relapse-free survival, suggesting that this 5′–C–phosphate–G–3′ (CpG) site may be crucial for TIM-1 production. Signaling by T cell receptor (TCRs) and CD28 is enhanced by TIM-1 co-stimulation to promote T cell proliferation and cytokine generation. TIM-1 is a potent co-stimulatory molecule for T cells (Umetsu et al., 2005; Freeman et al., 2010). TIM-1 is expressed differently in renal cell carcinoma (RCC) (McGregor et al., 2020), human colorectal cancer (CRC) (Wang et al., 2013), gastric cancer (Xue et al., 2019) and clear cell renal cell carcinoma (Cuadros et al., 2014), TIM-1 may have a direct role in tumour growth. Thus, we propose that TIM-1 predominantly influences tumour formation via two mechanisms: direct pathway (expressed in tumours) and indirect pathway (expressed in immune cells).

## 2 Molecular structure of TIM-1 and its ligands

### 2.1 Molecular structure

The TIM family of proteins are type-I membrane proteins, TIM-1 has an N-terminal immunoglobulin (Ig)V domain, a short cytoplasmic tail, a stalk domain with numerous potential O-linked glycosylation sites, a transmembrane domain, and a mucin-like domain with numerous potential N-linked glycosylation sites (Feigelstock et al., 1998). With the exception of TIM-4, all TIM molecules have a C-terminal cytoplasmic tail that contains a conserved tyrosine-based signaling motif. The TIM–IgV structural domain has high sequence homology (∼50%), but the mucin structural domain is highly diverse. The CD3–TCR complex transports a significant amount of intracellular TIM-1 to the immunological synapse where it co-localizes with the central supramolecular activation cluster. In contrast, cell-surface TIM-1is dispersed and does not interact with immunological synapses. Different subcellular locations of TIM-1 determine the bipolar sorting that is seen during the development of immunological synapses, which may control immune responses triggered by antigens (Echbarthi et al., 2015).

### 2.2 Ligands of TIM-1

TIM-1 binds mainly with two molecules,TIM-4 (Freeman et al., 2010) and phosphatidylserine (PS) (Angiari et al., 2014), but can also bind with P-selectin,S-selectin (Angiari et al., 2014), and the hepatitis A virus (HAV) (McIntire et al., 2001). PS is a phospholipid found on the outer leaflet of apoptotic cell membranes. It is a ligand for all members of the TIM family and can indicate cell death (Angiari et al., 2014). The PS molecule binds to the TIM-1 receptor present on the surface of natural killer T (NKT) cells to activate them (Echbarthi et al., 2015; Du et al., 2016). TIM-4 is a natural ligand for TIM-1, TIM-4 is expressed only on antigen-presenting cells and causes the phagocytosis of apoptotic cells. TIM-4 is crucial for maintaining tolerance (Freeman et al., 2010). The contact between TIM-1 and TIM-4 is through PS, which acts a bridge and stimulates the activation and proliferation of T cell and cytokine production (Meyers et al., 2005; Miyanishi et al., 2007; Xiao et al., 2011a). PS can bind to TIM-1 and TIM-4 specifically, enhance the interaction between TIM-1 and TIM-4, mediate apoptosis and activate NKT cells (Lee et al., 2010; Jemielity et al., 2013). P-selectin is a putative TIM-1 ligand. P-selectin is in charge of platelet binding and leukocyte recruitment during inflammatory reactions and autoimmune disorders. Adherence of primary T cells to P-selectin in living organisms is now dependent on TIM-1 as well as P-selectin glycoprotein ligand 1(PSGL-1) (Shamay et al., 2016). In a competitive binding mode with other TIM-1 ligands, HAV (which is an exogenous TIM-1 ligand) binds to TIM-1 expressed on Th2 cells. This action can inhibit activation of Th2 cells and affect T cell differentiation, leading to an imbalance between Th1 cell and Th2 cells (McIntire et al., 2001). IgA is a naturally occurring ligand for TIM-1 and their binding enhances the interaction between HAV and the receptor (Tami et al., 2007). In conclusion, for most of the time, TIM-1 can work only if it is bound to its ligand.

## 3 Possible pathways of action in TIM-1-mediated tumourigenesis and tumour development

### 3.1 Direct pathway

**FIGURE 1:**
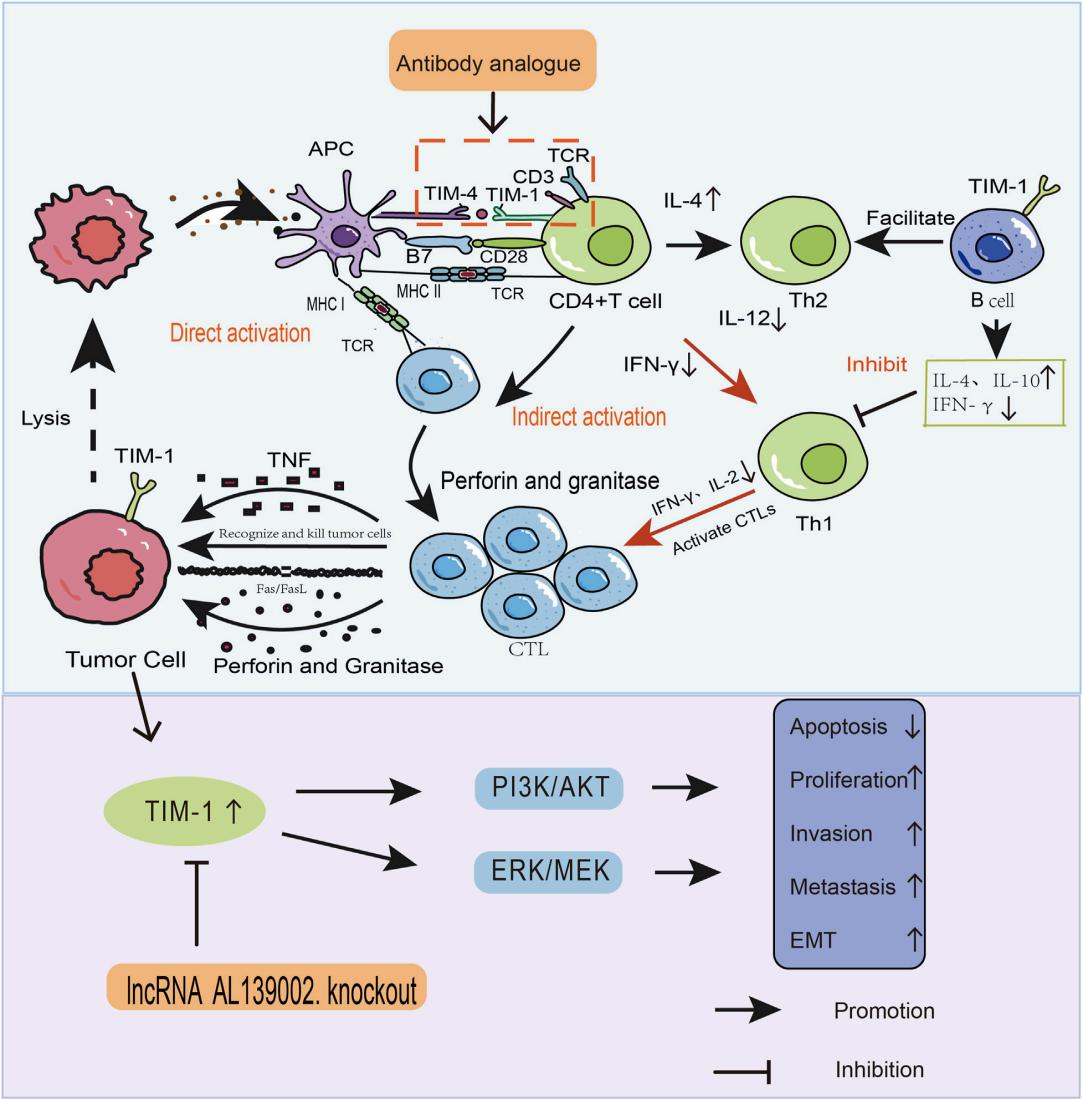
How TIM-1 affects the development and progression of tumours through two pathways. (1) Direct pathway. TIM-1 overexpression in tumours promotes the migration and invasion of tumour cells through MEK/ERK and PI3K/AKT signaling pathways. (2) Indirect pathway. In addition to mediating the onset and progression of tumours, TIM-1 binds to ligands, alters the tumour microenvironment, and stimulates the activation and proliferation of immune cells as well as cytokine production. These two pathways are entry points to design drugs that interfere with TIM-1 and related signaling pathways. This strategy can be used for tumour therapy.

TIM-1 is overexpressed in human CRC (Wang et al., 2013), RCC(McGregor et al., 2020), gastric cancer (Xue et al., 2019), Glioma (Zhang and Chen, 2022) and clear cell renal cell carcinoma (Cuadros et al., 2014). TIM-1 overexpression promotes tumour cell proliferation, inhibits apoptosis, mediates invasion and metastasis, and induces epithelial–mesenchymal transition EMT, which can promote tumour development directly. TIM-1 promotes the migration and invasion of tumour cells mainly through tumour the closely related pathways of mitogen-activated protein kinase/extracellular signal-regulated kinase (MEK/ERK) and phosphoinositide 3-kinase/protein kinase B (PI3K/AKT) (Figure 1) (Chen et al., 2022).

TIM-1 can regulate EMT and metastasis through MEK/ERK signaling (Xue et al., 2019; Chen and Zhang, 2021). Downregulation of TIM-1 expression inhibits the proliferation, colony formation, migration, and invasion of tumour cells by mediating activation of the MEK/ERK pathway (Santarpia et al., 2012; Xue et al., 2019). The RAF-MEK-ERK cascade is a major pathway of RAS-induced carcinogenesis. The RAS-RAF-MEK-ERK- MAPK (RAS-MAPK) signaling pathway is involved in regulating the growth, differentiation, proliferation, apoptosis, and migration of cells in tumourigenesis. ERK can be activated by various oncogenes and extracellular stimuli. ERK is phosphorylated by activated RAF upon RAS activation. Then, ERK translocates to the nucleus where it phosphorylates and controls a number of nuclear and cytoplasmic substrates involved in the proliferation, survival, differentiation, motility of cells, and angiogenesis (Santarpia et al., 2012; Xue et al., 2019). Downregulation of TIM-1 expression reduces the expression of phosphorylated MEK/ERK, which blocks activation of the MEK/ERK pathway and inhibit the proliferation, migration and invasion of tumour cells (Xue et al., 2019).

TIM-1 activates the PI3K/AKT/p53 and PI3K/AKT/mammalian target of rapamycin (mTOR) signaling pathways, which affects the cycle, cell growth, differentiation, survival, apoptosis, metabolism, and migration of cells, and angiogenesis. These changes can lead to EMT (Bray et al., 2018). These effects are closely related to protein expression levels, and it has been shown that TIM-1 is associated with downregulation of expression p53 and upregulation of expression of cyclin D1, which affects cell cycle and promotes the proliferation of tumour cell (Angiari et al., 2014). In addition, upregulation of TIM-1 expression controls the pathogenesis and progression of cancer by regulating activation of p53 and mTOR, downregulating expression of B-cell lymphoma-2 (Bcl-2)-associated X-protein (BAX), upregulating expression of Bcl-2, and inhibiting apoptosis in cancer cells (Banno et al., 2020). TIM-1 upregulates protein expression of Snail1, N-cadherin, Wiskott–Aldrich syndrome protein-family verprolin-homologous protein (waveform protein), matrix metalloproteinase-2 (MMP-2), and vascular endothelial growth factor (VEGF) in tumour cells and downregulates E-cadherin expression, allowing tumour cells to acquire stronger invasive and metastatic capacities (Shamay et al., 2016; Bray et al., 2018; Chen and Zhang, 2021). Studies have shown that targeted silencing of TIM-1 expression inhibits cancer cell growth *in vitro* and *in vivo* (Tami et al., 2007; Shamay et al., 2016). Overall, TIM-1 can directly affect the proliferation, invasion, and metastasis of tumour cells through MEK/ERK and PI3K/AKT pathways. Therefore, TIM-1 might be a biological target for cancers in therapeutic and anti-metastatic aspects.

### 3.2 Indirect pathways

Adoption of an anti-tumour phenotype by immune cells, contributes to immune surveillance and prevents tumour progression (Peña-Romero and Orenes-Piñero, 2022). Recent research has revealed that TIM-1 is primarily expressed on the surfaces of CD4^+^ T cells, CD8^+^ T cells, NK cells, macrophages, DCs, B cells, and mast cells (Li et al., 2013b). TIM-1 is also expressed in lymphoid organs (Rodriguez-Manzanet et al., 2009; Hu et al., 2011) and it can stimulate T cell responses by inducing cytokines and enhancing their production (Umetsu et al., 2005; Sizing et al., 2007; Curtiss et al., 2012; Lin et al., 2012; Schweigert et al., 2014). The primary factors governing immunological control by TIM-1 are its ligands (Rodriguez-Manzanet et al., 2009). PS and TIM-4 are the primary ligands of TIM-1 (Freeman et al., 2010; Angiari et al., 2014). By attaching to TIM-1 (which mediates the positive regulation of T cells and stimulates the immune response through a costimulatory action), TIM-4 can enhance the activation, proliferation, and cytokine production of T cells, all of which are essential for tumour immunity (Meyers et al., 2005; Miyanishi et al., 2007; Xiao et al., 2011a). TIM-4 and TIM-1 bind specifically to PS on the surface of apoptotic cells, but not to any other type of phospholipid tested so far (Ji et al., 2014). TIM-4^+^ peritoneal macrophages, TIM-1^+^ kidney cells, as well as TIM-4- or TIM-1-transfected cells phagocytose apoptotic cells efficiently, and phagocytosis can be blocked by monoclonal antibodies against TIM-4 or TIM-1 (Ji et al., 2014). TIM proteins have a unique binding cavity created by an unusual conformation of the CC’ and FG loops of the IgV domain of TIM, and mutations in this cavity eliminate PS binding and phagocytosis (Ji et al., 2014). Monoclonal antibodies against TIM-4 that block PS binding and phagocytosis map to epitopes in this binding cavity. TIM-4 and TIM-1 are immunologically restricted members of a group of receptors that recognize PS. These receptors are crucial for the efficient clearance of apoptotic cells, and may be essential for the maintenance of homeostasis and immune regulation during cancer development (Ji et al., 2014).

TIM-1 is also expressed on B cells (Wong et al., 2010). The different signals to T cells and B cells are important in maintaining normal homeostasis of the immune system. When immune cells adopt an anti-tumour phenotype, it contributes to immune surveillance and prevents tumour progression. TIM-1^+^ B cells promote the production of Th2 cytokines, interleukin (IL-4), IL-10, while inhibiting interferon (IFN)-γ production and suppressing Th1 immune responses (Thomas et al., 2016). Negative regulation of immune function of the TIM-1 in B cells also plays a key part in preventing immune rejection (Lee et al., 2012). Thus, TIM-1 is expressed on immune cells, regulates the tumour microenvironment, and influences tumour development indirectly by affecting tumour immunity.

#### 3.2.1 TIM-1 and T cells

The p85-adaptor component of PI3K is recruited by TIM-1-mediated T cell stimulation, which promotes T cell activation through the PI3K pathway. Tumour cells also produce signals that DCs take up. After being transformed into major histocompatibility complex (MHC) antigens, tumour antigens are given to the TCR for activation (de Souza et al., 2008). Upon polarization of CD4^+^ T cells, TIM-1 exists in CD4^+^ T cells (preferentially in Th2 cells). TIM-1 activation upregulates T cell activation and increases the expression of effector cytokines in CD4^+^ T cells, including Th2-type cytokines (IL-4, IL-5), and the T-regulatory cells (T_regs_)-inducible factor (IL-10) (Tan et al., 2014). These actions supply T cell costimulatory signals, boost the TCR and CD28 signaling involved in T cell proliferation/differentiation, and preventing the emergence of peripheral tolerance (Figure 1) (Umetsu et al., 2005; Liao et al., 2013). It is crucial for anti-tumour immunity that particular CD8^+^ T cells are cross-presented and cross-primed. They are the last line of defense against cancers, halting their progression *in vivo*. Cancer immunotherapy activates CD8^+^ T lymphocytes, which primarily use the perforin-granzyme and Fas/Fas-ligand-pathways to cause tumour cell apoptosis (Nowak et al., 2003; Wang et al., 2019). TIM-1 co-stimulatory signals may also enhance the role of CD8^+^ T cells (Nozaki et al., 2011). The binding of TIM-1 to its ligand alters CD4^+^ T cells, and CD4^+^forkhead box p3 (Foxp3)^+^ cells transforms into a CD4^+^ Th17^+^ phenotype, thereby promoting the differentiation of Th17 cells and reducing T_reg_ production (Degauque et al., 2008). T_regs_ can secrete the cytokines IL-10, and transforming growth factor (TGF-β), They contribute to the tumour *milieu* by suppressing the immune system (Thomas et al., 2016).

#### 3.2.2 TIM-1 and B cells

The homeostasis and the prevention of systemic autoimmunity of the immune system are dependent upon TIM-1 signaling on B cells ^[1]^. TIM-1^+^ B cells promote the production of the Th2 cytokines IL-4 and IL-10, while inhibiting IFN-γ production and suppressing Th1 immune responses (Figure 1) (Curtiss et al., 2012). By stimulating the growth of TIM-1^+^ B regulatory cells (B_regs_) cells and preventing the expression of cytokines such as IL-10, T cell immunoreceptor with Ig and ITIM domains (TIGIT), and inhibitory receptors, TIM-1 helps to induce and suppress B_regs_ function. Interactions between B_regs_ and T cells also reduce subsequent T cell interactions with DCs, enhance Th2 and T_reg_ (Foxp3, IL-10) responses, and suppress inflammatory responses by Th1 cells and Th17 cells responses (Figure 1) (Ji et al., 2014). It has been demonstrated that expB_reg_ express the CD73^−^CD25^+^CD71^+^ phenotype (similar to endogenous human IL-10^+^ B_reg_, and that TIM-1 and CD154 are required for expB_reg_ to have their suppressive effect. The suppressive power of human expB_reg_ cells can be increased by upregulating the phosphorylation of signal transducer and activator of transcription-3 (STAT3). Downregulating the function of TIM-1^+^ B_reg_ is a possible therapeutic target for tumour/immunological modulation. In particular, TIM-1 particularly positively regulates STAT3 phosphorylation (Shankar et al., 2022).

#### 3.2.3 TIM-1 and other types of immune cells

DCs cells have constitutive expression of TIM-1. TIM-1 signaling in DCs drives DC maturation by inducing nuclear factor-kappa B (NF-κB) activity, and its expression increases further after DC maturation. TIM-1 signaling increases the synthesis of pro-inflammatory cytokines and co-stimulatory molecules in DCs (but not in T cells), which encourages effector T cell responses. TIM-1 signaling also suppresses the creation of Foxp3^+^T_reg_ (Wong et al., 2010). TGF-β inhibits anti-tumour T cells and promotes the differentiation of Th17 cells (de Souza et al., 2008; Lee et al., 2012). The high affinity anti-TIM-1 monoclonal antibody 3B3 induces the activation of and increases NF-κB activity in DCs (Wong et al., 2010). As a result, TIM-1 is crucial for controlling DC activity and balancing effector T cell and T_regs_ to boost immunological responses (Wong et al., 2010). NK cells control tumour growth by releasing cytotoxic particles and death receptors to induce apoptosis in target cells (Liao et al., 2013). Phagocytes have tumouricidal effects and act as specialized antigen-presenting cell-activated T cells (Rodriguez-Manzanet et al., 2009). TIM-1 binding to TIM-4 on activated Th2 cells can directly activate macrophages, and TIM-1 on activated Th1 cells triggers IFN-γ production, which leads to indirect macrophage production (Ueno et al., 2008). Recent work revealed that TIM-1 activation increased expression of B7 family member and cytokine release in macrophages (Hein and Woods, 2007).

## 4 TIM-1 and tumours types

### 4.1 RCC

RCC is the third most prevalent urologic malignancy worldwide, causing 100,000 deaths each year. ccRCC is the most prevalent and aggressive subtype of RCC. More than 70% of patients with ccRCC or papillary renal cell carcinoma have TIM-1 detected in their tumour tissue. TIM-1 shows low expression in the normal renal cortex and in other solid tumours. The plasma level of TIM-1 can be used to predict RCC development. TIM-1 is a highly sensitive marker for the early diagnosis of ccRCC (Scelo et al., 2018; Kushlinskii et al., 2019; McGregor et al., 2020). TIM-1 expression is associated with a more malignant phenotype of RCC, and shedding of the outer structural domain is linked with tumour progression (McGregor et al., 2020). Clinical studies have shown TIM-1 expression: to be significantly higher than normal in the plasma of patients with benign and malignant renal tumours; increased significantly with disease stage; to be significantly lower in patients with benign tumours than in patients with malignant tumours (Mijuskovic et al., 2018; Kushlinskii et al., 2019). In ccRCC, *in vitro* experiments have shown that TIM-1 can induce IL-6 expression, thereby activating STAT-3, promoting angiogenesis through the IL-6/STAT-3 pathway, which is conducive to tumour growth and metastasis (Cuadros et al., 2014). Additional findings suggest that targeted silencing of TIM-1 expression inhibits the growth of ccRCC cells *in vitro* and *in vivo* (Tami et al., 2007). *In vitro* experimental studies have found that TIM-1 overexpression can block the differentiation of human ccRCC (769-P) cells, so TIM-1 may participate in cell dedifferentiation and tumourigenesis (Vilà et al., 2004). Overall, TIM-1 is a screening, diagnostic, and monitoring biomarker for renal cancer.

### 4.2 Hepatocellular carcinoma (HCC)

Hepatocellular carcinoma is the fourth leading cause of cancer-related deaths worldwide and its associated mortality rate is expected to rise within the next decade (Alawyia and Constantinou, 2023). One clinical study showed that the percentage of TIM-1^+^ B_reg_ was increased significantly in the HCC compared with that in blood and peri-cancer. Also microvascular infiltration, early recurrence, and the TNM stage were all positively connected with the proportion of TIM-1^+^ B_reg_ in tumour tissue (Ye et al., 2018). In patients with HCC, significant infiltration of TIM-1^+^ B cells were linked to advanced disease and a poor prognosis. An undiscovered fraction of tumourigenic B cells known as TIM-1^+^ B_regs_ cells had a CD5^high^CD24^−^CD27^−/+^CD38^+/high^ phenotype. Three main mechanisms that underpin the involvement of TIM-1^+^B_reg_ cells in HCC progression of HCC. First, ectoplasmic bodies released by HCC cells stimulate accumulation of the TIM-1^+^ B_regs_ cell accumulation through the high mobility group box 1 (HMGB1) -TLR2/4-MAPK pathway. Second, by secreting IL-10 and reducing the activity of CD8^+^ T cells, TIM-1^+^ B_regs_ cells provide an immunosuppressive milieu that facilitates HCC evolution. Third, via TIM-1/TIM-4 signaling, myeloid cells strengthen the immunosuppressive ability of TIM-1^+^ B_regs_. Consequently, treatments that block the HMGB1-TLR2/4-MAPK immunosuppressive pathway and TIM-1^+^ B_regs_ may be a unique approach to treating HCC (Ye et al., 2018).

### 4.3 Stomach cancer

Gastric cancer is the fifth leading cause of morbidity and mortality in prevalence worldwide (Ferlay et al., 2021; WHO, 2024). Gastrointestinal adenocarcinomas (GAC) accounts for ∼90% of stomach cancer cases (Karimi et al., 2014). mRNA and protein expression of TIM-1 has been shown to be much higher in GAC tissues than in adjacent normal tissues. HAVCR-1 might be a different predictor of the prognosis for stomach cancer if TCGA-STAD data are analyzed using univariate and multivariate Cox regression models (Liu et al., 2018). *In vitro* studies have shown that knockdown of HAVCR-1 expression on GAC cells can lower expression of phosphorylated (p)-MEK and p-ERK expression markedly, prevent the MEK/ERK pathway from being activated, and prevent the proliferation, colony formation, migration, and invasion of GAC cells (Xue et al., 2019). In addition, expression of the long-non-coding (lnc) RNA AL139002.1 has been found to be upregulated in GAC cells, and that lncRNA AL139002.1 binds competitively to microRNA-490-3p to regulate HAVCR-1. The latter has a carcinogenic role in the proliferation, apoptosis, migration and invasion of GAC cells through the MEK/ERK signaling pathway after upregulation (Chen and Zhang, 2021). The shedding of HAVCR-1 in gastric cancer increases the expression of IL-6 in RCC and activates the IL-6/STAT-3/HIF-1α pathway. This pathway mediates resistance to trastuzumab, EMT, and mutual crosstalk between cancer-derived mesenchymal stem cells and neutrophils, ultimately promoting the progression and metastasis of gastric cancer (Liu et al., 2018). Blockade of the interaction between TIM-1 and TIM-4 enhances the effect of DC vaccines against gastric cancer (Sun et al., 2012). Taken together, these data suggest that TIM-1 could be utilized as a predictive biomarker for stomach cancer, which could offer a fresh approach to researching the etiology of GAC.

### 4.4 CRC

CRC has the third highest incidence (1.9 million cases, 9.6%) and second highest mortality (900,000 deaths, 9.3%) in the worldwide (Sung et al., 2021; WHO, 2024). TIM-1 expression can increase the risk of *Helicobacter pylori* infection (Zhang et al., 2012). TIM-1 expression has been shown to be increased in clinical tissue samples of colon cancer, and the disease-free time of patients with high expression of TIM-1 was prolonged significantly. *In vitro* cell-function experiments showed that: TIM-1 overexpression had no effect on the growth of colon cancer cells (HRT18, Caco2, HT115) but could reduce the adhesion and invasion of colon cancer cells (Wang et al., 2013). The interaction between TIM-1 and TIM-4 could induce FasL expression in cancer cells, thereby inducing apoptosis. TIM-1 was expressed in human colon cancer cell lines (HT29, T84 cells). After TIM-4 treatment, TIM-1 was activated to increase the expression of Tip60, H3K9, RNA polymerase II significantly and, finally, activate STAT3 at the FasL promoter site, resulting in increased apoptosis frequency of colon cancer cells. TIM-4 induced apoptosis of cancer cells was also inhibited by blocking Fas–FasL interaction (Wang et al., 2016). Therefore, TIM-1 may be a novel therapeutic target for colon cancer, and TIM-1/TIM-4 can induce cancer cells to undergo apoptosis.

### 4.5 Lung cancer

In 2024, the World Health Organization’s International Agency for Research on Cancer (IARC) released the latest. Globally, there will be 20 million new cases of cancer-related and 9.7 million cancer deaths in 2022. Lung cancer was the most commonly occurring cancer worldwide with 2.5 million new cases accounting for 12.4% of the total new case (WHO, 2024). There are two types of lung cancer: non-small-cell lung cancer (NSCLC) and small-cell lung cancer (SCLC). Approximately 85% of cases of lung cancer are NSCLC (Skřičková et al., 2018). Histology of clinical samples revealed TIM-1 expression in NSCLC tissues to be increased compared with that in near-normal tissues, and that high expression of TIM-1 was closely associated with a poor prognosis of NSCLC (Zheng et al., 2019). Cox regression models showed that higher TIM-1 expression in lung-cancer tissues was an independent prognostic predictor (Zheng et al., 2019; Kong et al., 2020). TIM-1-mediated control of NSCLC function is regulated through the PI3K/AKT signaling pathway. *In vitro* cell investigations have demonstrated that inhibition of TIM-1 expression blunts the ability of A549 cells and SK-MES-1 cells to migrate and invade, respectively (Zheng et al., 2019). TIM-1 was favorably connected with CD56dim NK cells in lung cancer and had a negative relationship with the levels of NK cell, gamma delta T cells, and T_regs_ infiltration in lung cancer, according to a study on the association between the infiltration of immune cells and TIM-1 in malignancies (Kong et al., 2020). These investigations provide fresh insights into the pathophysiology of lung cancer and shed light on the function of TIM-1 in its progression.

### 4.6 Cervical cancer

Cervical cancer is the eighth most commonly occurring cancer globally and the ninth leading cause of cancer death, accounting for 661,044 new cases and 348,186 deaths (WHO, 2024). Among cancers that affect only women, cervical cancer has the fourth-highest incidence and fatality rate (Singh et al., 2022). Patients with distant metastases from cervical cancer have a dismal prognosis, and metastasis and recurrence account for most of the majority of deaths associated to cervical cancer (Singh et al., 2022). According to one clinical study, TIM-1 expression was increased in cervical-cancer tissues, whereas *in vitro* cell experiments indicated that TIM-1 reduced the expression of p53, BAX, and E-cadherin and increased the expression of VEGF, MMP-2, cyclin D1, Bcl-2, Snail1, N-cadherin, vimentin, and MMP-2. Total expression of AKT was constant, but protein expression of PI3K, p-AKT, and mTOR proteins increased. Overall, in cervical cancer, through controlling the PI3K/AKT/p53 and PI3K/AKT/mTOR signaling pathways, TIM-1 overexpression reduces apoptosis, promotes cell migration/invasion, and controls the cell cycle and proliferation (Chen et al., 2022). TIM-1 could be a useful biomarker and therapeutic target for the management of cervical cancer.

### 4.7 Glioma

Glioma is the most common malignant tumour in the brain, originating from the glial cells of the neuroectoderm. Its primary traits include a high incidence, high risk of death, and poor chance of cure, which are primarily elicited by the rapid growth of glioma (Gilard et al., 2021). Some clinical studies have shown that TIM-1 expression in glioma tissues is significantly higher than that in normal paraneoplastic tissues, and that the high expression of TIM-1 in gliomas correlates with the Karnofsky Performance Status score and histology grade. TIM-1 expression in glioma tissues can affect patient survival, and is an independent risk factor for gliomas (Zhang and Chen, 2022). Aberrant expression of TIM-1 is strongly associated with development of human gliomas through regulation of the PI3K/AKT pathway. *In vitro* experiments have demonstrated that knockdown of TIM-1 expression inhibited the proliferation, migration, and invasion of glioma (U87, U251) cells and decreased the levels of TGF-β1, IL-6, IL-4, and IL-10 in gliomas. A combination of an activator of the PI3K/AKT pathway and knockdown of *Tim-1* expression partially reversed these results. After deletion of TIM-1, the volume and weight of the tumour and prevalence of Ki67-positivity decreased in nude mice (Zhou et al., 2020). TIM-1 may be a potential therapeutic target for gliomas.

### 4.8 TIM-1 and other tumours

Primary central nervous system lymphoma (PCNSL) is an uncommon form of extranodal non-Hodgkin’s lymphoma that arises within the central nervous system (Deckert et al., 2011). Some studies have found that TIM-1 has high expression in PCNSL according to histology of clinical specimens and the Gene Expression Omnibus database, and that TIM-1 can promote IL-10 production in PCNSL cells. Detection of soluble TIM-1 in cerebrospinal fluid may be helpful in the diagnosis and evaluation of PCNSL (Kishimoto et al., 2016).

Breast cancer is the most common malignancy in women worldwide. Even though the prevalence of mortality of breast cancer has decreased as a result of early detection and improved treatment, it is the leading cause of cancer-related death in women (Sung et al., 2021). One study demonstrated higher expression of neutrophil gelatinase-associated lipocalin and TIM-1 in breast cancer with *in situ* components (Diniz et al., 2022). Cai et al. (2021) developed a high-powered predictive classifier for the diagnosis of breast cancer via eight mRNAs from plasma extracellular vesicles (including TIM-1), which could provide a new approach for the early diagnosis of breast cancer. The study also showed that the relevance of interrogating the levels of TIM-1 protein as a diagnostic/prognostic biomarker of high-prevalence breast and lung cancers by using an amperometric disposable magnetic microparticles-assisted immunoplatform (Quinchia et al., 2024).

An uncommon malignant tumour known as Langerhans cell sarcoma develops from epidermal DCs and is distinguished by cellular heterogeneity, numerous mitotic divisions, and aggressive clinical behavior. According to morphological analyses, TIM-1 is expressed in pathological sections of Langerhans cell sarcoma. Cells that express TIM-1 include sarcomatoid cells, CD68^+^ macrophages, infiltrating inflammatory cells, and CK-18^+^ epithelial cells (Li et al., 2013a). TIM-1 expression could aid in the creation of a novel immunotherapeutic or diagnostic approach for Langerhans cell sarcoma.

## 5 TIM-1 and tumour treatment

Based on literature review and our previous study, we hypothesize that two pathways of TIM-1-mediated tumour development are the entry points for tumour therapy. Drugs are designed to interfere with TIM-1 and related signaling pathways to inhibit the invasion and metastasis of tumour cells directly. The other, design agonistic antibodies against the costimulatory receptor TIM-1 for tumour immunotherapy; By blocking the TIM-1 signaling pathway, regulating T cell activation, promoting anti-tumour immunity in the body, and controlling tumour growth.

### 5.1 Tumour therapy based on a direct pathway

Metastasis is the leading cause of cancer-related deaths. Cancer drugs have been developed for nearly one century, but OS at 5 years of patients with metastatic cancer (especially those with distal metastases) is low. Efficacious therapeutic strategies that target metastases specifically are scarce (Steeg, 2016; Esposito et al., 2021). The main therapeutic options for cancer metastasis include inhibiting neoangiogenesis, EMT blockade, and searching for metastasis suppressors (Steeg, 2016). Moreover, studies have shown EMT to be associated with tumour invasion, metastasis formation, and treatment resistance (Lüönd et al., 2021). Recent studies have shown that TIM-1 has high expression in solid tumour tissues (Cuadros et al., 2014; Xue et al., 2019; McGregor et al., 2020), and that its expression is closely related to the invasion and metastasis abilities of tumours. *In vitro* and *in vivo* experiments have shown that TIM-1 can affect the proliferation, invasion, and metastasis of tumour cells directly through MEK/ERK and PI3K/AKT pathways (Xue et al., 2019; Zheng et al., 2019; Zhou et al., 2020; Chen et al., 2022). TIM-1 activates PI3K/AKT/p53 and PI3K/AKT/mTOR signaling pathways, affects the cycle, growth, differentiation, apoptosis, metabolism, and migration of cells, and angiogenesis, and these changes may lead to EMT (Chen et al., 2022). TIM-1 might be a therapeutic and anti-metastatic target of cancer. A recent study may have demonstrated the feasibility of this strategy. Thomas et al. (Thomas et al., 2016). Developed a novel antibody–drug conjugate (ADC) that targets tumour cells expressing TIM-1A. Human IgG1κ monoclonal antibody specific for TIM-1 is bonded covalently to the enzyme-cleavable microtubule inhibitor vcMMAE to form an ADC: CDX-014. *In vitro*, the ADC was shown to exhibit cytostatic or cytotoxic activity against human TIM-1-expressing cell lines [lung squamous carcinoma (A549), ovarian adenocarcinoma (IGROV-1), ccRCC (Caki-1)] (Thomas et al., 2016). Using Caki-1, IGROV-1, and A549 xenograft mouse models, CDX-014 showed significant antitumour activity in a clinically relevant dose range (Thomas et al., 2016). In 2020, the first clinical trial of CDX-014 evaluated its safety and preliminary activity, and demonstrated a new approach to treating refractory RCC (McGregor et al., 2020). Hence, inhibiting TIM-1 expression directly, regulating downstream signaling pathways (e.g., MEK/ERK, PI3K/AKT), inhibiting the proliferation, invasion, and metastasis of tumour cells, and thereby controlling tumour growth are rational approaches for tumour treatment.

### 5.2 Tumour therapy based on an indirect pathway

A vital part of cell-mediated immunity against tumours is the type-1 immune response, which is mediated by Th1 cells, cytotoxic T lymphocytes (CTLs), NK cells, NKT cells, and gamma delta T cells (Coe et al., 2013; Jiang et al., 2013). Th1 cells and CTLs in tumours can be a positive prognostic factor in humans (Galon et al., 2006). A different approach to cancer therapy is to increase the T cell-mediated immune response of a patient by utilizing agonist antibodies recognizing some members of the TNFR family that activate T cells. These include CD40 GITR, OX40, and 4-1BB (Redman et al., 2015; Djureinovic et al., 2021; Jhajj et al., 2023). These therapeutic approaches are being tested in clinical trials (some of them with promising results). As a co-stimulatory molecule, TIM-1 is expressed mainly on the surface of T cells, but also on the surface of B cells, DCs, macrophages, NKT cells, and other lymphocytes (Du et al., 2016; Kong et al., 2020). Thus, agonistic antibodies against co-stimulatory receptors such as TIM-1 have shown promising antitumour effects in various studies. TIM-1-Ig affects TIM-1–TIM-4 interactions *in vivo*, leading to T cell hyperactivation, allowing them to continue proliferating *in vitro* and producing Th1 (IL-2, IFN-γ) and Th2 (IL-4, IL-10) cytokines. TIM-4–Ig induces the hyperproliferation of T cells *in vivo* and co-stimulation of T cell proliferation *in vitro* (Meyers et al., 2005). Two anti-TIM-1 antibodies, RMT1-10 and 3B3, differing by 17-fold in binding affinity to the same or closely related Tim-1 epitope in the IgV domain, exert opposite effects on T cell function (Xiao et al., 2007). A mouse model of ovalbumin-induced pulmonary inflammation showed that a TIM-1-activating antibody (3B3) promoted the proliferation of CD4^+^ T cells and IL-4 secretion by Th2 cells, but did not have a significant effect on Th1 cell-derived IFN-γ expression (Umetsu et al., 2005; Hu et al., 2011). Isolation of an *in vitro* culture of C57BL/6J lymphocytes revealed that stimulation of 3B3 anti-TIM-1 monoclonal antibody enhanced polarization of Th1 cells/Th17 cells and increased the expansion and survival of CD4^+^ and CD8^+^ alloreactive cells *in vitro* (Degauque et al., 2008). Tan et al. used a mouse model of high-risk corneal transplantation to determine the effect of systemic administration of RMT1-10. This can be achieved by inhibiting the activation and proliferation of CD4^+^ T cells, as well as inhibiting the alloreactive Th-cell response while enhancing the T_regs_ response (Tan et al., 2014). In mouse EAE models and *in vitro* cell experiments revealed that use of high-affinity TIM-1 agonist antibodies increased the immunogenic function of DCs and increased the pro-inflammatory Th17 response *in vivo*, shifting the balance between effector T cells and T_regs_ towards an enhanced immune response (Xiao et al., 2011b). Studies have shown that blockade of the TIM-1 signal can reduce the number of CD8^+^ T cells significantly and inhibit IFN-γ secretion in a cisplatin-induced model of acute renal injury, suggesting that a TIM-1 co-stimulative signal can enhance the role of CD8^+^ T cells (Nozaki et al., 2011). This study suggested that targeting TIM-1 molecules could regulate the proliferation and differentiation of lymphocytes and regulate the immune response. Therefore, the co-stimulatory molecule TIM-1 could provide a new target for tumour induction and enhancement of an effective endogenous immune response. Hence, targeted intervention using the TIM-1 molecule may be a new approach for anti-tumour therapy.

TIM-4 binds specifically to TIM-1, regulates the activation and proliferation of T cells, and phosphorylates the downstream T cell signals AKT and ERK, thereby playing an important part in mediating the phagocytosis of apoptotic cells (Tami et al., 2007; Su et al., 2008). Experimentation with tumour therapy could involve blockade of the interactions between TIM-1 and TIM-4. TIM-1 and TIM-4 recognize PS on apoptotic cells and function as co-stimulators (Freeman et al., 2010; Dayoub and Brekken, 2020). PS signaling is exploited by tumours to enhance immune evasion, so strategies to inhibit PS-mediated immune suppression could increase the efficacy of immunotherapy (Dayoub and Brekken, 2020). Tumour therapy could involve blockade of the interactions between TIM-1 and TIM-4. The small interfering (si)RNA of the FG-CC’ gene can block the FG-CC’ ring. This can be achieved by transfecting the siRNA of the FG-CC’ gene into the DC stimulated by gastric cancer lyase, and FG-CC-siRNA plays an important part in the interaction between *TIM-1* and *TIM-4*. The action between TIM-1 and TIM-4 is blocked, so the effect of the DC vaccine on gastric cancer is enhanced (Sun et al., 2012). *In vitro* cell-function experiments showed that FasL expression in cancer cells could be induced by activating the TIM-1−TIM-4 interaction after TIM-4 treatment, thus increasing the prevalence of apoptosis of colon cancer cells. TIM-4-induced apoptosis of cancer cells was also inhibited by knocking out TIM-1 expression and blockade of Fas–FasL interactions (Wang et al., 2016). Therefore, regulating the interaction between TIM-1 and TIM-4, mediating tumour-cell apoptosis, and controlling the occurrence and development of tumours may be a new approach for tumour treatment.

## 6 Conclusion and future perspectives

TIM-1 is a key member of the TIM family and a co-stimulatory molecule. TIM-1 is expressed differentially in different immune cells, tumour tissues, and cell surfaces. TIM-1 has complex and important roles in tumour immunity and is involved in disease development (especially in the etiology of tumours). We hypothesize that the targeted intervention of TIM-1 may be a new type of anti-tumour therapy. This intervention could be achieved by designing excitatory antibodies against TIM-1 for inhibiting tumour proliferation, preventing EMT occurrence, invasion and metastasis of cells, promoting apoptosis, and controlling tumour growth directly. Another approach could be to block its signaling pathway, prevent its interaction with corresponding ligands, regulate the function of immune cells, and control the occurrence and development of tumours through immunotherapy.

In summary, TIM-1 may be a new target for tumour treatment. Designing specific antibodies and/or small-molecule compounds for tumour treatment could be a very promising strategy.
